# Incidence Hypertension and Fasting Blood Glucose from Real-World Data: Retrospective Cohort for 7-Years Follow-Up

**DOI:** 10.3390/ijerph18042085

**Published:** 2021-02-21

**Authors:** Soon-Ki Ahn, Ju-Mi Lee, Seon Mi Ji, Kyoung Hoon Kim, Jong-Heon Park, Min Kyung Hyun

**Affiliations:** 1 Public Health and Medical Services Office, Chungnam National University Hospital, Jung-gu, Daejeon 35015, Korea; withspirit09@gmail.com; 2 Department of Preventive Medicine, Eulji College of Medicine, Daejeon 34824, Korea; aqualeo0731@gmail.com; 3 National Health Insurance Service, Wonju 26464, Korea; parkjh@nhis.or.kr; 4 Health Insurance Review & Assessment Service, Wonju 26465, Korea; rudqnr@hanmail.net; 5 Department of Preventive Medicine, College of Korean Medicine, Dongguk University, Gyeongju-si 38066, Korea; mk3three@dongguk.ac.kr

**Keywords:** incidence, hypertension, blood glucose, prediabetic state, prehypertension

## Abstract

This retrospective cohort study was done to investigate the incidence of hypertension and its relation to the fasting blood glucose level in Korea. The eligible non-hypertensive subjects (*n* = 3,396,187) among the National Health Insurance Service-National Health Screening (NHIS-HEALS) examinees (*n* = 10,644,911) in 2009 were followed up until 2015. A Cox proportional hazards regression was used to estimate the risk of the high blood glucose level for the incident hypertension while controlling for covariates’ confounding effect. The cumulative incidence rate was 10.6% for seven years (11.6% in men and 8.3% in women). The incidence density was 1474.8 per 100,000 person-years. High fasting blood glucose (adjusted Hazard Ratio (aHR), 1.836; 95% confidence interval (CI), 1.810 to 1.862), prediabetes (aHR, 1.249; 95% CI, 1.237 to 1.260), a history of diabetes mellitus (aHR, 1.635; 95% CI, 1.605 to 1.666), high triglyceride (aHR, 1.292; 95% CI, 1.280 to 1.303), a history of dyslipidemia (aHR, 1.279; 95% CI, 1.253 to 1.305) and prehypertension group (aHR, 1.964; 95% CI, 1.948 to 1.979) were significantly related to the incident hypertension after adjusting for covariates. Among real-world data in Korea, high blood glucose level was the independent risk factor for developing hypertension.

## 1. Introduction

Hypertension is the third leading risk factor for the global burden of disease and the leading risk factor for cardiovascular and kidney disease [1,2,3,4]. About 54% of stroke and 47% of ischemic heart disease worldwide were attributable to high blood pressure [5]. The estimated total number of adults with hypertension in 2000 was 972 million (957–987 million), and it was projected to increase by 60% to a total of 1.56 billion (1.54 billion–1.58 billion) in 2025 worldwide [6]. According to the 2018 Health Insurance Statistical Yearbook, there are 6.31 million hypertensive patients in Korea that use 333.29 billion won for medical expenses, and patients increased by 4.2% compared to the previous year and 7.1% the prior year [7]. Hypertension is two times more common among patients with diabetes than those without diabetes, so strict blood pressure control needs to be a high priority in caring for patients with type 2 diabetes [8].

Meanwhile, prediabetes is defined as the presence of IFG (impaired fasting glucose) or IGT (impaired glucose tolerance) or raised HbA1c (5.7–6.4% by ADA 2010 criteria). Prediabetes is not only a clinical entity in itself, it should be considered an increased risk of diabetes and cardiovascular disease (CVD) [9,10]. However, evidence is less clear regarding hypertension mediating the causative relationship between prediabetes and CVD [11]. Prediabetes and hypertension induce endothelial dysfunction and inflammation by elevating levels of soluble adhesion molecules and inflammatory cytokines. The comorbidity of these diseases may exacerbate inflammation and endothelial dysfunction [12]. According to the American Heart Association (AHA), lifestyle factors such as body mass index, physical activity, diet, and cigarette smoking are critical determinants of blood pressure levels [13,14]. Several factors contribute to an accelerated diabetes epidemic, including the obesity phenotype; high prevalence of smoking and heavy alcohol use; high intake of refined carbohydrates; and dramatically decreased physical activity levels [15]. 

Fasting plasma glucose in the prediabetes range is independently and significantly associated with the future development of hypertension [16]. It is essential to pay attention to the early stage of hypertension and diabetes, to control the transition from prehypertension and prediabetes to hypertension and diabetes [17]. Although many studies of prevalence and incidence of hypertension have been conducted in various sample sizes, areas or workplaces, and age groups [18,19], there is limited research on prediabetes on development of hypertension with a nationwide scale in Korea.

Therefore, this study was conducted to investigate the incidence of hypertension and its relation to the fasting glucose level among non-hypertensive national health examinees in Korea.

## 2. Materials and Methods

### 2.1. Data Source

Korean National Health Insurance Service (NHIS) is the only insurer, covering approximately 96.6% of the entire South Korean population, and has a claim database and general health examination database. This retrospective observational cohort study used the National Health Screening (HEALS) and NHIS database. The HEALS Program invites individuals to participate at least once every 2 years in a general free-of-charge health screening program. HEALS and NHIS databases are linked to personal identification numbers and anonymized and provided to a designated secure computer in the security room.

The National Health Insurance Service-National Health Screening (NHIS-HEALS) database includes medical diagnoses, drug prescriptions, demographic characteristics, and information from health examinations, such as self-administered health surveys, physical examinations, and biochemical test results. Details of the cohort have been published previously [20,21,22,23].

### 2.2. Study Design

Briefly, study candidates were 10,644,911 individuals aged over 19 years old who participated in HEALS between 1 January 2009, and 31 December 2009. Due to the study aim, individuals were excluded if they had baseline (2009) blood pressure (BP) values of ≥140 mmHg systolic or ≥90 mmHg diastolic, or a diagnosis of hypertension (International Classification of Disease-Tenth Revision, Clinical Modification Code (ICD-10-CM:) I10, 11, 12, 13, 15). After that, all participants were categorized in normal or high-normal blood pressure categories based on the national health examination in 2009. 

**Figure 1:**
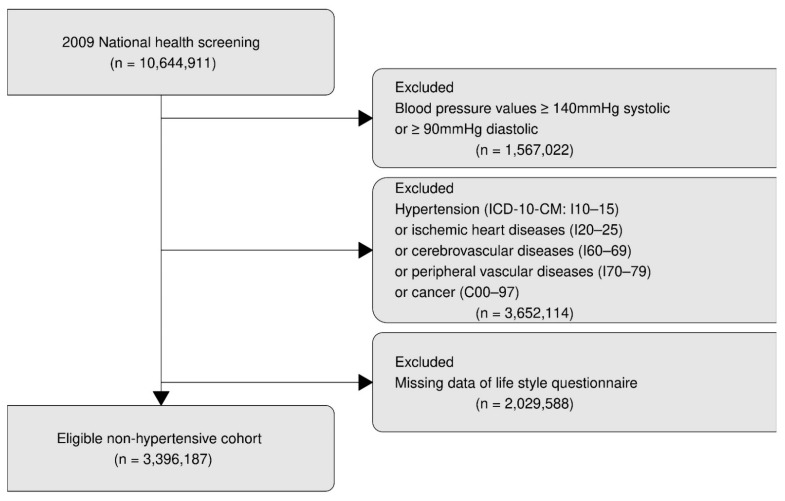
Flowchart of study participants.

Persons with a history of ischemic heart diseases (ICD-10-CM: I20 to 25), cerebrovascular (ICD-10-CM: I60 to 69), or other peripheral vascular diseases (ICD-10-CM: I70 to 79) were excluded because of the possible effect of such diseases on accurate blood pressure. Persons with a history of malignancy (ICD-10-CM: C00 to 97) and missing data which did not meet the inclusion criteria were excluded (Figure 1). Finally, 3,396,187 non-hypertensive subjects were our retrospective cohort participants and followed up for seven years until 2015.

### 2.3. Outcome Variable

The primary endpoint was identified based on ICD-10-CM codes (I10, I11, I12, I13, and I15) in the medical record from the NHIS database.

### 2.4. Independent Variables

Blood samples were taken using a venipuncture during the health examination after an overnight fast of at least 8 h. Total cholesterol, serum glucose, triglycerides (TG), and high-density lipoprotein (HDL) cholesterol were enzymatically assessed. Body mass index (BMI) was estimated as body weight (kg) divided by height squared (m^2^). 

Study subjects were classified by measured fasting blood glucose levels into 3 groups (≥126 mg/dL; 100–125 mg/dL; <100 mg/dL) based on a classification and diagnosis of diabetes using the standards of medical care in diabetes of the American Diabetes Association (ADA) in 2018 [9]. For the present study, prehypertension was identified as systolic blood pressure (BP) levels in the range of 120 to 139 mmHg or diastolic BP between 80 and 89 mmHg, according to the Seventh Joint National Committee on the Prevention, Detection, Evaluation, and Treatment of Hypertension (JNC-VII) [24].

Metabolic syndrome was defined in subjects with three or more of the following criteria: (1) Abdominal obesity, waist circumference ≥90 cm in men or ≥80 cm in women; (2) Hypertriglyceridemia, TG ≥150 mg/dL or medication use; (3) Low HDL–cholesterol, HDL-cholesterol <40 mg/dL in men and <50 mg/dL in women; (4) High systolic blood pressure (BP), systolic BP ≥130 mmHg and/or diastolic BP ≥85 mmHg; (5) Hyperglycemia, fasting blood glucose >100 mg/dL or use [25]. 

Current smokers were identified as those who had smoked more than 100 cigarettes in their lives or were currently smoking. High-risk drinking was defined as drinking more than 300 mL per day on average. For traditional Korean drinks, one standard drink unit corresponds to one bowl (approximately 300 mL) of Korean rice beer (Makgeolli) or a quarter bottle (approximately 90 mL) of 20% Korean liquor (Soju) [26]. The optimal exercise was identified as follows: (1) intensive exercise lasting more than 20 min per session and more than three times per week or (2) moderate exercise lasting more than 30 min per session and more than five times per week. Household income was calculated based on the insurance owner’s income level to claim health insurance premiums and was classified into quintiles.

### 2.5. Statistical Analysis

The continuous variables were reported as the mean value ± standard deviation (SD) and compared using a Student t-test. The categorical variables were presented as the frequency and proportion (%) and compared using a chi-squared test. The incidence density of newly diagnosed hypertension per 100,000 person-years (PY) was calculated as the number of participants who developed new onset of hypertension during the follow-up period divided by the sum of individual follow-up periods of those at risk. 

Multicollinearity among covariates was evaluated by assessing deviations of regression coefficients and their standard errors (SEs) in the fitted multivariate models, but none were detected.

Cox proportional hazards regression was used to estimate the prognostic influence of non-hypertension on conversion to hypertension while simultaneously controlling for covariates’ confounding effects. Non-hypertensive subjects were divided into two groups for the multivariate proportional hazard regression models: the normotensive and the pre-hypertensive. The adjusted hazard ratio (aHR) and 95% confidence interval (CI) are reported. A two-sided probability value less than 0.05 were considered statistically significant. Data was analyzed with SAS statistical software, version 9.4, for Windows (SAS, Cary, NC) and R software (version 4.0.2, the R Foundation for Statistical Computing, Vienna, Austria, http://www.r-project.org). 

## 3. Results

### 3.1. Baseline Characteristics of the Study Participants

The baseline characteristics of the study participants are shown in Table 1. Among the 3,396,187 total subjects, the proportion of men was 70.7%. Excluding the metabolic syndrome variable, all the proportions of the other variables were statistically significantly higher in men than in women (*p* < 0.001).

The mean age was 38.1 ± 11.0 years old (38.6 ± 10.8 years in men and 37.0 ± 11.5 years in women). The mean BMI was 23.3 ± 3.1 kg/m2 (23.8 ± 3.0 years for men and 22.1 ± 3.1 years for women).

The proportion of prediabetes with fasting blood glucose levels between 100–125 mg/dL was 23.8% in men and 14.7% in women, and that with high fasting blood glucose levels ≧126 mg/dL, 3.9% in men, and 1.5% in women.

The proportion of systolic blood pressure levels between 120–139 mm Hg was 59.8% in men and 33.5% in women, and that with diastolic blood pressure level between 80–89 mm Hg was 43.9% in men and 22.7% in women. History of diabetes mellitus was 2.0% in men and 0.7% in women, and history of dyslipidemia was 2.3% in men and 0.9 % in women.

In addition, 54.8% of men, and 6.3% of women subjects were categorized as current smokers. High risk drinking was assessed in 16.4% of men and 3.1% in women. Optimal exercise was assessed in 18.7% of men and 13.7% of women. The highest household income level was 23.0% in men and 13.7% in women.

The proportion of metabolic syndrome was 52.2% in men and 78.6% in women (*p* < 0.001).

### 3.2. Baseline Characteristics between the New-Onset Hypertension and Non-Hypertensive Group with the Cumulative Incidence

Table 2 shows the baseline characteristics between the new-onset hypertension and non-hypertensive group with the cumulative incidence. During the study, 360,346 new cases of hypertension were recorded among 3,396,187 participants. The cumulative incidence rate was 10.6% for 7 years (11.6% in men and 8.3% in women). The mean age of the new-onset hypertension cases was 46.4 ± 11.5 years old. The cumulative incidence rate of prediabetes was 14.4%, high fasting blood glucose was 29.0%, systolic blood pressure level between 120–139 mm Hg was 14.7%, and diastolic blood pressure level 80–89 mm Hg was 15.8%. The cumulative incidence rate of history of diabetes mellitus was 29.6%, history of dyslipidemia was 18.5 %, metabolic syndrome was 8.0%, lowest house income level was 11.3%, and highest house income level was 12.9%.

### 3.3. Incidence Density of New Incident Hypertension

Table 3 shows the incidence density of new incident hypertension was 1474.8 per 100,000 person-year (PY). Accordingly, the calculated total observation years were 21,037,305.5 PY. Within our observation period, 360,346 subjects were diagnosed with new incident hypertension.

**Figure 2:**
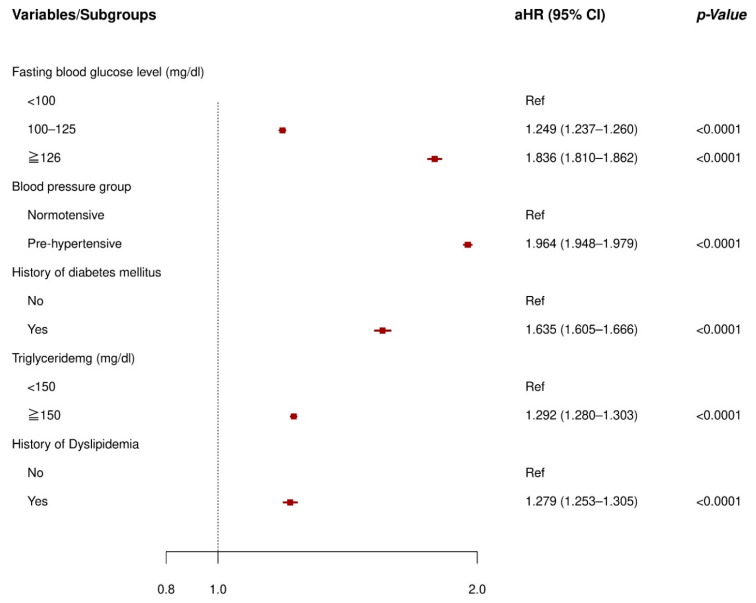
Forest plot of the adjusted Hazard ratio of incident hypertension. Abbreviations: aHR = adjusted Hazard Ratio; CI = Confidence Interval; Ref = Reference. Adjusted for age, sex, body mass index, waist circumference, high-density lipoprotein cholesterol, metabolic syndrome, current smoking, high risk alcohol drinking, optimal exercise, and household income.

The multivariate Cox regression analysis for identifying independent risk factors of newly diagnosed hypertension and its forest plot is presented in Figure 2. In the multivariate model, high fasting blood glucose (adjusted Hazard Ratio (aHR), 1.836; 95% confidence interval (CI), 1.810 to 1.862), prediabetes (aHR, 1.249; 95% CI, 1.237 to 1.260), a history of diabetes mellitus (aHR, 1.635; 95% CI, 1.605 to 1.666), high triglyceride (aHR, 1.292; 95% CI, 1.280 to 1.303), a history of dyslipidemia (aHR, 1.279; 95% CI, 1.253 to 1.305), and prehypertension group (aHR, 1.964; 95% CI, 1.948 to 1.979) were significantly related to the incident hypertension after adjusting for age, sex, body mass index, waist circumference, HDL-cholesterol, metabolic syndrome, current smoking, high-risk alcohol drinking, optimal exercise, and household income (Supplementary Table S1).

## 4. Discussion

Our study result showed that high blood glucose levels, such as diabetes level and prediabetic level, were independent risk factors for new-onset hypertension in the National Health Screening examinees in Korea.

During the follow-up study, new-onset hypertension was found in 360,346 people. The cumulative incidence rate was 10.6% over seven years (11.6% for men and 8.3% for women) at the mean age of 38. The incidence density of new-onset hypertension was 1474.8 per 100,000 person-years. Compared to the previous studies, one prospective cohort study reported that the cumulative incidence of hypertension in middle-aged Koreans was 17.3%. This difference can be explained by the fact that the study subjects’ average age was 50.5 years, which was higher than 38.1 years of age in this study. Like our study, one real-world claimed data study reported that the incidence density of hypertension in middle-aged Koreans was about 50 per 1000 people [27]. This difference can be explained by the fact that the study subjects were 40 years of age or older, which was higher than our study subjects’ age.

In 1967, one case-controlled (people with diabetes and an equal number of controls) study showed the prevalence of hypertension was 54% greater among the people with diabetes [28]. Since then, many studies reported comorbid status and the association between diabetes mellitus and hypertension. Approximately 75% of patients with diabetes had concomitant hypertension, and both conditions also increased with age [29]. Recently, diabetes mellitus was considered a risk factor for hypertension. One study showed diabetes mellitus at baseline was a significant predictor of incident hypertension [30]. All of these results are consistent with our results.

Possible mechanisms in developing hypertension in diabetes mellitus have been continuously suggested. Diabetes and hypertension share common pathways such as the sympathetic nervous system (SNS), renin-angiotensin-aldosterone system (RAAS), oxidative stress, adipokines, insulin resistance, and Peroxisome proliferator-activated receptors (PPARs) [31]. These pathways interact and influence each other. For example, insulin resistance causes hyperglycemia, dyslipidemia, and hyperinsulinemia, leading to vascular smooth muscle cell proliferation, increasing arterial stiffness, vascular tone, sodium retention, sympathetic nervous system, and decreasing vasodilation, thus leading to high blood pressure.

Preclinical stages were associated with new-onset hypertension. This study was consistent with the results of fasting plasma glucose in the prediabetes range, which is independently and significantly associated with future development of hypertension among large scale nationwide research subjects in Korea [18]. In the Korean Genome and Epidemiology Study (KoGES), which followed 10,038 patients for ten years, the incidence of hypertension in the prediabetes group was insignificant compared to the normoglycemia group [11]. In contrast, in the present study, the risk of hypertension was 1.8 times higher in the high fasting blood glucose group and 1.2 times higher in the prediabetes group. The prehypertensive group had about two times the incidence of hypertension than the normotensive group, which was the most potent risk factor in the present study. The previous study reported 2–3 times [27,32], perhaps due to the age difference. Our result is consistent with previous studies [33,34,35]. Age, sex, body mass index, waist circumference, metabolic syndrome, current smoking, high-risk alcohol drinking, and household income were generally consistent with other studies, but high-density lipoprotein cholesterol and optimal exercise were not. Inconsistent results seem to be the effect of differences in distribution by gender and age. The new-onset hypertension group was more likely to be comprised of female, older, prediabetes, the high fasting blood glucose, history of diabetes mellitus, dyslipidemia, history of dyslipidemia, metabolic syndrome, current smoking, high-risk drinking, and lower household income participants, compared to the non-hypertensive group.

In the present study, diet and water contained minerals were not considered. Higher sodium intake and lower potassium intake are known to play an important role in the development of hypertension [14,36,37]. According to previous studies, differences in the age-adjusted prevalence of hypertension across racial and ethnic groups were reported, in the order of non-Hispanic black, non-Hispanic white, Hispanic, and non-Hispanic Asian [14,38,39,40], but the difference could not be confirmed in the present study because Korea was composed of a single ethnic group.

Our study has several strengths. Our study showed clear insight that diabetes and prediabetes range of blood glucose level are risk factors for developing hypertension with real-world data. Second, our study has the strength of being of large scale with nationwide research subjects, making it easier to generalize the research results. Third, our study design has advantages, such as not being interrupted with recall bias and selective reporting reduction. Fourth, the final model considered AHA’s simple seven healthy behavior and social-economic status by adjusting age, sex, blood pressure level, lipid profiles, house income, smoking, drinking, and exercise.

The present study has some limitations. First, we identified the hypertension cases using only claims data, including diagnostic and treatment codes, and could not validate the diagnosis of hypertension through direct review of medical records, which is an inherent limitation of studies using the claim database. Second, due to data limitation, we could not analyze the changes in health status and lifestyle during the follow-up period, and the prediabetes group was defined as just only fasting blood glucose level. Third, there were about 2.5 times fewer female samples than males, and gender stratification could not be performed.

## 5. Conclusions

Our study confirmed real-world data that high blood glucose levels were an independent risk factor for developing hypertension among non-hypertensive national health examinees in Korea. This study suggested meaningful insight because it is consistent with other studies’ results: well-performed cohort study results and case-controlled study results. This study may present an opportunity to reform the community-based programs for prevention and management that have been in progress in about 250 local health centers in Korea for more than several decades.

A further study may be required to establish cut-off levels for optimal glucose levels for developing incident hypertension. Additionally, laboratory investigations may be required to understand the exact roles of each stage of the pathophysiologic mechanisms in the development of hypertension at a high glucose level.

## Figures and Tables

**Table 1:** Baseline characteristics of study participants.

Variables/Subgroups	Total (*n* = 3,396,187)	Men (*n* = 2,399,713, 70.7%)	Women (*n* = 996,474, 29.3%)	*p*-Value
Age (years) (Mean ± SD)	38.1 ± 11.0	38.6 ± 10.8	37.0 ± 11.5	<0.0001
Fasting blood glucose level (mg/dL)				
<100	2,570,560 (75.7)	1,734,786 (72.3)	835,774 (83.9)	<0.0001
100–125	717,924 (21.1)	571,748 (23.8)	146,176 (14.7)	
≧126	107,703 (3.2)	93,179 (3.9)	14,524 (1.5)	
Systolic BP (mmHg)				
<120	1,626,329 (47.9)	963,848 (40.2)	662,481 (66.5)	<0.0001
120–139	1,769,858 (52.1)	1,435,865 (59.8)	333,993 (33.5)	
Diastolic BP (mmHg)				
<80	2,115,361 (62.3)	1,345,291 (56.1)	770,070 (77.3)	<0.0001
80–89	1,280,826 (37.7)	1,054,422 (43.9)	226,404 (22.7)	
BMI (kg/m^2^) (Mean ± SD)	23.3 ± 3.1	23.8 ± 3.0	22.1 ± 3.1	<0.0001
Waist circumference (cm)				
Men <90, Women <85	2,944,248 (86.7)	2,025,580 (84.4)	918,668 (92.2)	<0.0001
Men ≧90, Women ≧85	451,939 (13.3)	374,133 (15.6)	77,806 (7.8)	
Triglyceride (mg/dL)				
<150	2,436,931 (71.8)	1,542,728 (64.3)	894,203 (89.7)	<0.0001
≧150	959,256 (28.3)	856,985 (35.7)	102,271 (10.3)	
HDL (mg/dL)				
Men <40, Women <50	470,338 (13.9)	287,031 (12.0)	183,307 (18.4)	<0.0001
Men ≧40, Women ≧50	2,925,849 (86.2)	2,112,682 (88.0)	813,167 (81.6)	
History of diabetes mellitus				
No	3,341,335 (98.4)	2,351,550 (98.0)	989,785 (99.3)	<0.0001
Yes	54,852 (1.6)	48,163 (2.0)	6689 (0.7)	
History of dyslipidemia				
No	3,332,463 (98.1)	2,345,162 (97.7)	987,301 (99.1)	<0.0001
Yes	63,724 (1.9)	54,551 (2.3)	9173 (0.9)	
Metabolic syndrome				
No	1,360,773 (40.1)	1,147,127 (47.8)	213,646 (21.4)	<0.0001
Yes	2,035,414 (59.9)	1,252,586 (52.2)	782,828 (78.6)	
Current smoking				
No	2,018,092 (59.4)	1,084,540 (45.2)	933,552 (93.7)	<0.0001
Yes	1,378,095 (40.6)	1,315,173 (54.8)	62,922 (6.3)	
High risk alcohol drinking				
No	2,972,682 (87.5)	2,006,597 (83.6)	966,085 (97)	<0.0001
Yes	423,505 (12.5)	393,116 (16.4)	30,389 (3.1)	
Optimal exercise				
No	2,812,478 (82.8)	1,952,078 (81.4)	860,400 (86.3)	<0.0001
Yes	583,709 (17.2)	447,635 (18.7)	136,074 (13.7)	
Household income				
1Q (lowest)	519,078 (15.3)	314,804 (13.1)	204,274 (20.5)	<0.0001
2Q	618,641 (18.2)	366,173 (15.3)	252,468 (25.3)	
3Q	767,447 (22.6)	543,158 (22.6)	224,289 (22.5)	
4Q	801,814 (23.6)	623,335 (26)	178,479 (17.9)	
5Q (highest)	689,207 (20.3)	552,243 (23)	136,964 (13.7)	

Abbreviations: BP = Blood Pressure; BMI = Body Mass Index; HDL, High-Density Lipoprotein cholesterol.

**Table 2:** Baseline characteristics between new-onset hypertension and non-hypertensive group with the cumulative incidence.

Variables/Subgroups	New-Onset (*n* = 360,346, 10.6%)	Undiagnosed (*n* = 3,035,841, 89.4%)	*p*-Value
Sex			
Men	277,760 (11.6)	2,121,953 (88.4)	<0.0001
Women	82,586 (8.3)	913,888 (91.7)	
Age (years) (Mean ± SD)	46.4 ± 11.5	37.1 ± 10.5	<0.0001
Fasting blood glucose level (mg/dL)			
<100	225,930 (8.8)	2,344,630 (91.2)	<0.0001
100–125	103,161 (14.4)	614,763 (85.6)	
≧126	31,255 (29.0)	76,448 (71.0)	
Systolic BP (mmHg)			
<120	99,746 (6.1)	1,526,583 (93.9)	<0.0001
120–139	260,600 (14.7)	1,509,258 (85.3)	
Diastolic BP (mmHg)			
<80	158,271 (7.5)	1,957,090 (92.5)	<0.0001
80–89	202,075 (15.8)	1,078,751 (84.2)	
BMI (kg/m^2^) (Mean ± SD)	24.5 ± 3.1	23.2 ± 3.1	<0.0001
Waist circumference (cm)			
Men <90, Women <85	273,573 (9.3)	2,670,675 (90.7)	<0.0001
Men ≧90, Women ≧85	86,773 (19.2)	365,166 (80.8)	
Triglyceride (mg/dL)			
<150	215,102 (8.8)	2,221,829 (91.2)	<0.0001
≧150	145,244 (15.1)	814,012 (84.9)	
HDL (mg/dL)			
Men <40, Women <50	60,753 (12.9)	409,585 (87.1)	<0.0001
Men ≧40, Women ≧50	299,593 (10.2)	2,626,256 (89.8)	
History of diabetes mellitus			
No	344,095 (10.3)	2,997,240 (89.7)	<0.0001
Yes	16,251 (29.6)	38,601 (70.4)	
History of Dyslipidemia			
No	348,566 (10.5)	2,983,897 (89.5)	<0.0001
Yes	11,780 (18.5)	51,944 (81.5)	
Metabolic syndrome			
No	196,822 (14.5)	1,163,951 (85.5)	<0.0001
Yes	163,524 (8.0)	1,871,890 (92.0)	
Current smoking			
No	211,367 (10.5)	1,806,725 (89.5)	<0.0001
Yes	148,979 (10.8)	1,229,116 (89.2)	
High risk alcohol drinking			
No	299,332 (10.1)	2,673,350 (89.9)	<0.0001
Yes	61,014 (14.4)	362,491 (85.6)	
Optimal exercise			
No	287,381 (10.2)	2,525,097 (89.8)	<0.0001
Yes	72,965 (12.5)	510,744 (87.5)	
Household income			
1Q (lowest)	58,639 (11.3)	460,439 (88.7)	<0.0001
2Q	56,659 (9.2)	561,982 (90.8)	
3Q	71,786 (9.4)	695,661 (90.7)	
4Q	84,722 (10.6)	717,092 (89.4)	
5Q (highest)	88,540 (12.9)	600,667 (87.2)	

Abbreviations: BP = blood pressure; BMI = body mass index; HDL = high-density lipoprotein cholesterol.

**Table 3:** Incidence density of new-onset hypertension by year.

Follow-Up Period (Years)	Population at Risk (n)	New-Onset Hypertension (n, %)	Censored (n)	PY	Incidence (per 100,000 PY)
0–1	3,396,187	51,247 (1.51)	2620	3,394,877	1509.0
1–2	3,342,320	55,942 (1.67)	3066	3,340,787	1647.8
2–3	3,283,312	57,250 (1.74)	3437	3,281,593.5	1713.7
3–4	3,222,625	56,043 (1.74)	3418	3,220,916	1707.8
4–5	3,163,164	56,460 (1.78)	3247	3,161,540.5	1752.9
5–6	3,103,457	56,420 (1.82)	2876	3,102,019	1784.6
6–7	3,044,161	26,984 (0.89)	3,017,177	1,535,572.5	869.9
total	3,396,187	360,346 (10.61)	3,035,841	21,037,305.5	1474.8

Abbreviations: PY = Person-Year.

## Data Availability

Because of the policy of National Health Insurance Service of Korea, dataset is not permitted to be taken out of the NHIS. The data can be accessed on the National Health Insurance Data Sharing Service homepage of the NHIS (http://nhiss.nhis.or.kr). Applications to use the NHIS-HEALS data will be reviewed by the inquiry committee of research support, and once approved, raw data will be provided to the applicant with a fee. Although the datasets are coded in English and numbers, not in Korean (Hangul), the use of individual data is allowed only for Korean researchers. But it would be possible for researchers outside the country to gain access to the data by conducting a joint study with Korean researchers.

## Supplementary Materials

The following are available online at https://www.mdpi.com/1660-4601/18/4/2085/s1, Supplementary Table S1: Adjusted Hazard ratio of incident hypertension by baseline characteristics.
