# Supplementary material for: Incidence Hypertension and Fasting Blood Glucose from Real-World Data: Retrospective Cohort for 7-Years Follow-Up
Source: Int J Environ Res Public Health. 2021 Feb 21;18(4):2085. doi: 10.3390/ijerph18042085 (PMC7924835; doi:10.3390/ijerph18042085)
Supplement: Supplementary file 1 [file ijerph-18-02085-s001.pdf]

Supplementary Table S1. Adjusted Hazard ratio of incident hypertension by baseline characteristics.

Variables	aHR	95%CI	*p-Value*
**Sex**			
Men	Ref		
Women	1.059	1.049–1.069	<0.0001
Age (years)	1.063	1.063–1.063	<0.0001
Fasting blood glucose level (mg/dl)			
<100	Ref		
100–125	1.249	1.237–1.260	<0.0001
≧126	1.836	1.810–1.862	<0.0001
Blood pressure group			
Normotensive	Ref		
Pre-hypertensive	1.964	1.948–1.979	<0.0001
BMI (kg/m^2)	1.091	1.089–1.092	<0.0001
Waist circumference(cm)			
Men <90, Women <85	Ref		
Men ≧90, Women ≧85	1.174	1.162–1.186	<0.0001
Triglyceride (mg/dl)			
<150	Ref		
≧150	1.292	1.280–1.303	<0.0001
HDL (mg/dl)			
Men <40, Women <50	Ref		
Men ≧40, Women ≧50	1.046	1.036–1.057	<0.0001
History of diabetes mellitus			
No	Ref		
Yes	1.635	1.605–1.666	<0.0001
History of Dyslipidemia			
No	Ref		
Yes	1.279	1.253–1.305	<0.0001
Metabolic syndrome			
No	Ref		
Yes	1.208	1.195–1.222	<0.0001
Current smoking			
No	Ref		
Yes	1.076	1.068–1.084	<0.0001
High risk alcohol drinking			
No	Ref		
Yes	1.203	1.192–1.214	<0.0001
Optimal exercise			
No	Ref		
Yes	1.015	1.007–1.024	0.0002
Household income			
1Q (lowest)	Ref		
2Q	0.985	0.973–0.996	0.0087
3Q	0.976	0.966–0.987	<0.0001
4Q	0.961	0.951–0.971	<0.0001
5Q (highest)	0.911	0.902–0.921	<0.0001

Abbreviations: BMI = body mass index; HDL = high-density lipoprotein cholesterol; aHR = adjusted Hazard Ratio; CI = Confidence Interval; Ref = Reference.
